# The tenth Janet Doe Lecture, a forty-year perspective: still relevant after all these years

**DOI:** 10.5195/jmla.2020.864

**Published:** 2020-01-01

**Authors:** Wayne J. Peay, Helen-Ann Brown Epstein

**Affiliations:** Librarian Emeritus, Spencer S. Eccles Health Sciences Library, University of Utah, Salt Lake City, UT, waynejpeay@gmail.com; Informationist, Health Sciences Library, Virtua Health, Mt. Laurel, NJ, hepstein@virtua.org

## Abstract

Erich Meyerhoff was an academic health sciences librarian and a distinguished member of the Medical Library Association when he was invited to present the Janet Doe Lecture in 1977. His lecture on the state of the association is considered one of the finest Doe lectures and is still relevant more than forty years later, not only from an historical perspective, but also for his projections for the future and his prescient comments about the future of hospital librarianship and the important role of women in the association. Key 1977 Doe lecture topics are reviewed and updated in the context of the current health sciences library environment.

## INTRODUCTION

In 1966, the Janet Doe Lecture was established by an anonymous donation in her honor and continues to be a featured highlight at each annual meeting of the Medical Library Association (MLA). Erich Meyerhoff presented the tenth lecture at the annual meeting in 1977 [1]. Prior lectures addressed a diversity of topics, depending on the lecturer’s interest. Erich was the first to survey the state of the profession and to offer projections for the future. Lucretia W. McClure described this lecture as “a valuable history of MLA advances and changes [in] the early days of automation. It is worth reading again and again” [2]. The objectives of this essay are to briefly review the topics surveyed in 1977 and provide updates forty years later.

## RESOURCES AND SERVICES

Not surprisingly, the initial focus of the lecture was library resources, and Erich begins with the observation that “The growth and development of resources, technology, and services in all medical libraries is an astonishing achievement.” At the time, he reported on the significant improvement of medical school library budgets with median budgets of $328,093 in 1973/74, up from $57,471 in 1960/61. It is worth noting that the current median budget, as reported in the Association of Academic Health Sciences Libraries (AAHSL) 41st annual survey in 2019, is $3,367,407 which, adjusted for inflation, is a 41% increase [3]. The number of journal subscriptions was the standard measure at the time for libraries. The journal subscription median was 1,942 in 1975, compared to 992 in 1960. With regret, he noted that the goals of collection development were being frustrated by “Inflation, the devaluation of the dollar and the sharp curtailment of funds in the health sector of our economy [that] suddenly put a halt to the euphoric hopes of continuous resource development.” While the issues he described were, in part, limiting factors, it turns out that the explosive growth of the literature, digital resources requiring restrictive licenses, and exploitative publishers have all contributed to constraining access to resources.

Erich described the emergence of the interlibrary loan (ILL) and document delivery services supported by the Regional Medical Library Network that provided an essential support for hospitals and an offset to the cost constraints. Erich quoted a 1975/76 survey that recorded nearly 925,000 ILLs [4]. This compares to 492,842 ILLs reported in the 2018 AAHSL annual survey. While the current activity is impressive, the significant difference can be attributed to the loss of ILL subsidies, the increase in hospital libraries, and the impact of technology. DOCLINE 6.0 was introduced in summer 2019 on an open source platform. Libraries have set up cells of lenders to receive quick responses first from neighbors and ultimately the National Library of Medicine. Many of these transactions are free of charge; however, some libraries do still charge for ILLs. The emergence of ILL services that Erich noted marked a major advance in the evolution of libraries from information sources to information services.

Virginia Algermissen and Gertrude Lamb developed clinical librarian programs at the University of Missouri School of Medicine, described as a model for cultivating the relationship between librarians and members of an interprofessional team by delivering information services at the point of need on the spot or in a timely manner. Clinical librarianship, covered only briefly in the 1977 lecture, has continued to develop and proved to be flexible enough to succeed in a variety of environments, such as bedside rounds and morning report. The emergence of the informationist role in health care, described by Frank Davidoff and Valerie Florance, has shown that this model can succeed as presented from the perspective of the specialty of informatics [5]. While there may be differences in terminology, the goals of providing much needed information in the clinical setting remain challenging.

Erich noted the beginning of the new and exciting dimension of library-based patient education programs. Today, some libraries have added a service for patients and their families with easier-to-understand lay medical information or a separate patient education or consumer health library. These libraries provide information so that patients can be informed consumers of health care, may offer access to the electronic medical record, and may be staffed by a librarian.

## EDUCATION

At the time of the lecture, library education was entering a serious downturn. Specialty programs such as medical librarianship were being abandoned and intern programs were closing, as were entire academic library programs. Certainly, Erich felt these losses more than most since the highly regarded medical bibliography course that he taught at the Columbia School of Library Service was ended, and subsequently, the entire school was closed. This was the fate of other library schools across the country, but there has been a remarkable turnaround made possible by the Internet and distance education. Initially viewed with skepticism, the 2019 American Library Association (ALA)–accredited library and information sciences program lists 49 schools that offer 100% online programs, 13 schools offer selected online courses, and 8 do not offer anything online [6]. Erich cited Julie Virgo’s full-dress review of medical library education in 1975 listing 62 accredited library schools, with 37 offering medical library education. The 2017 *US News & World Report* reported 51 library schools, with only 7 offering health sciences librarianship [7]. With many librarians finding their way into the profession by first working in libraries, this access to advanced degrees is a significant advantage that, in the past, involved the time and expense of relocating to attend on-campus programs.

Contributing to the lecture’s discouraged perspective on education was the loss of federal funding for training and internship programs. Erich stated, “discontinuance is a political fact and a contemporary tragedy.” These programs offered advanced instruction in computer applications, administration, and other medical library specialties. In fact, a study for the highly regarded Graduate School of Library and Information Sciences, University of Illinois–Urbana-Champaign, noted that in the late twentieth century more subject specialists with doctorates from outside the field of library and information science (LIS) were hired as LIS faculty, contributing to the multidisciplinarity of the field. The study questioned whether this was going to be a trend in LIS professional education that would result in the disintegration of the LIS discipline [8].

Fortunately, the education partnership of the academic health sciences community and the National Library of Medicine (NLM) has been revived with the establishment of the NLM/AAHSL Leadership Fellows Program. Spurred by an informal survey that reported 50% of the library directors would retire by 2010 and 75% by 2015, AAHSL formed the Future Leadership Task Force that developed a program plan resulting in the fellows program and securing NLM support [9]. The program included a recruitment guide, an introductory MLA continuing education (CE) course, scholarships for leadership activities, and mentoring with a current director. To date, 82 fellows have participated in the program, and nearly half have been appointed as library directors [10].

With library schools closing or no longer offering courses in medical librarianship, MLA has assumed a greater responsibility for professional continuing education. Courses are taught on the local, regional, or national level. MLA has a CE clearinghouse website, MEDLIB-ED, with course offerings. In 2018, MLA created subcommittees of the education committee charged with designing the basis of hour-long self-paced online learning opportunities. Taking the outline of the committees, a subject specialist, an instructional designer, and the MLA education director will produce the courses. An overarching committee has been also been formed to choose CE for the MLA annual meeting and the roster of webinars for the coming year. This work continues to face the challenge of education for medical librarians posed by Estelle Brodman in 1954 and Erich in 1977: “‘[It] is not the *how* but the *why* of medical librarianship’…What appears to emerge as the nexus of our concern is communication, information, and its mode of transfer.”

## TECHNOLOGY AND RESEARCH

In Erich’s long career and life, he witnessed, certainly with some satisfaction, the early transformation from paper to the digital environment that is now the world of libraries. The 1970s were a decade of transition, with beginning of the decade witnessing the first applications of computing, based on the room-sized mainframes managing data input and data stored on punch cards. By the middle of the decade, mini-computers were appearing the size of washing machines, and magnetic disk drives were capable of storing data in the single digits of megabytes. Finally, at the end of the decade, the personal computer, at significant cost, began the true transformation of libraries.

Erich noted the important introduction of these technologies with the innovative work at the University of California Los Angeles (UCLA) and Washington University. His library at Cornell Medical College was an early adopter of the Washington University serials management system, PHILSOM. He also referred to the innovative application of data networking provided by the Ohio College Library Center (OCLC) online cataloging system. He noted the efficiencies derived from shared library systems in reducing the “clerical routine.” However, the real power of this service turned out to be the data network. Facilitating collaboration that was seen in the shared cataloging services was only a hint of what the technology had to offer.

At the time of the lecture, ARPANET had been developing for nearly a decade, and its utility would spin-off similar networks: NSFNET, CSNET, and BITNET. As the transmission-control protocol (TCP)/Internet protocol (IP) emerged, it provided the foundation that allowed the various networks to merge into what is now the Internet. In what may be the most significant technical and cultural development of the century, the Internet has provided libraries with an unprecedented research and development environment. The next generation of the Internet is on our doorstep with the promise of very high-speed networking, pervasive access, and an exciting environment to pursue the continuing development of library resources and services that Erich envisioned that would bring information to one’s fingertips.

At various points in the lecture, Erich described multiple instances of essential NLM support for libraries. He also addressed in detail the efforts by libraries to develop effective systems of classification that led to NLM’s development of Medical Subject Headings (MeSH), which set a standard for libraries and for the Internet. In these early days of the Internet, there was frequently the lament, “if only the Internet could function as well as libraries.” Erich accurately observed that “The organization of knowledge in the health sciences, however, remains an important and unresolved problem, worthy of best minds and the best efforts.” This continues to be an essential challenge for our profession as can be seen NLM’s Strategic Plan for 2017 to 2027: “By increasing the speed at which information is organized, disseminated, and accessed, NLM will accelerate the speed and precision of discovery” [11].

## HOSPITAL LIBRARIES

The 1990 Doe Lecture by Ruth Holst, AHIP, FMLA, traced the history of hospital librarianship with some personal reflection. She referenced Erich’s 1977 Janet Doe Lecture: “In his closing remarks, he speculated that ‘the emergence of hospital librarians as a creative and productive group of practitioners with professional strivings and close relationships with their clientele represents a pool of talent which has already begun to make its mark’” [12].

His speculation was spot on. The decade of the 1970s was characterized by growth for individual hospital libraries, as well as a sense of coming together in networks and consortia that strengthened the process of resource sharing to put librarians closer in touch with their colleagues in other basic unit libraries. During the 1980s, the climate changed. The hospital industry was competing in the business marketplace and felt the need to adopt a more businesslike approach, which often times required hospital librarians to defend the hospital library against hospital economic cutbacks and to adopt a businesslike attitude for the hospital library.

Adaptation to the new environment was assisted by the development of a four-part online CE course and subsequent publications. In June 2013, members of the Hospital Library Advisory Committee of the Middle Atlantic Region of the National Network of Libraries of Medicine debuted a network-funded four-part online CE course: Running Your Hospital Library Like a Business: “Session 1, A Paradigm Shift: Asking ‘Why’ Before Saying ‘Yes’”; “Session 2, Writing a Business Plan”; “Session 3, The Art of Negotiation”; and “Session 4, Proving Your Worth/Adding to Your Value.” Incorporated into session 4 were summaries of three hallmark hospital library studies: the King Study, the Rochester Study, and the Values Study [13].

In 1984, the US Health Care Financing Administration (HCFA) eliminated the requirement that hospitals maintain a library in order to qualify for Medicare reimbursement. The goal of the King Study was to “assess the ability of the hospital library to deliver, in a timely fashion, published information and library services which may be of value for clinical care” [14]. The main takeaway from the King Study was that most of the health professionals surveyed were satisfied with the overall performance of their libraries in meeting their clinical needs.

In 1988, in a move similar to the HCFA requirement, the New York State Department of Health eliminated the requirement for a health sciences library, stating that the department could not find a “useful linkage” between the hospital maintaining a health sciences library and any problems that occurred with the delivery of hospital patient care and services. The Rochester Study confirmed the findings of the King Study that information provided by hospital librarians was perceived by physicians as having a significant impact on clinical decision-making and that the increased use of such information could help reduce the frequency and severity of adverse events in hospitalized patients [15].

In the early 2000s, hospital administrators questioned the expense of library resources and the librarian’s salary, using the rationalization that the Internet and Google could provide all needed information. Hospital libraries were downsized or closed, and hospital librarians had their hours reduced or they were let go. In 2005, MLA created the Task Force on Vital Pathways for Hospital Librarians to develop an action plan for MLA to influence the hospital decision-makers about the library. A website, white papers, PowerPoint slides, brochures, an advocacy toolkit, and a series of journal articles in the October 2009 issue of the *Journal of the Medical Library Association (JMLA)* were published to arm the hospital librarian with supporting evidence that the hospital librarian and hospital library were essential to fulfilling a hospital’s goal to deliver quality clinical care, education, innovation, research, and customer service [16].

The results of the Value of Library and Information in Patient Care Study (Values Study) were published in 2013. The study replicated the landmark Rochester Study but expanded the libraries studied to the Middle Atlantic Region of the National Network of Libraries of Medicine [17]. The results strongly suggested that the hospital library and the information resources provided by hospital librarians had a valuable impact on patient care.

The MLA presidential goals of M.J. Tooey, AHIP, FMLA, in 2005 and Dixie Jones, AHIP, in 2013, and the Doe lectures by Erich, Holst, and Margaret Bandy, AHIP, FMLA, memorialized hospital librarians as a creative and productive group of practitioners with professional strivings and close relationships with their clientele [12, 18]. Bandy concluded that hospital librarians who develop interprofessional and collaborative relationships and provide relevant services would demonstrate the hospital librarian as a vital contributing member of the health care organization.

Hospital librarians are a creative pool of talent as Erich predicted in 1977, but the economics of health care continue to influence hospital librarians and hospital libraries. The new Hospital Libraries Caucus of MLA is again surveying members and gathering benchmarking data about the status of hospital libraries and librarians and their valuable contributions to patient care, education, and research efforts. Advocacy and convincing marketing materials are being created to distribute to influential decision makers.

## WOMEN IN MEDICAL LIBRARIANSHIP

Erich noted medical librarianship was overwhelmingly composed of women. Today, it remains a female-dominated profession. Women have made noteworthy contributions to our profession. Since 1977, all but seven presidents of MLA have been women and all but twenty members of the MLA Board of Directors have been women. At this writing, the current Board of Directors is all women. More women than men have delivered the Doe lecture. Other notable women leaders include Carla J. Funk, CAE, retired long-term executive director of MLA, and Patricia Flatley Brennan, NLM director and a nurse informatician. Comparing the 1977 report by Rachael K. Goldstein and Dorothy R. Hill with women holding 39% of head librarian positions [19] to the 2019 AAHSL directory [20], women members now constitute 76% of directors.

## CONCLUSION

Doe lectures are a highlight of each MLA annual meeting and certainly a milestone in the career of the presenter. In most instances, the lecture focuses on a topic of importance to the lecturer and often provides revealing insights into the lecturer as well. Erich’s lecture was unique because he addressed not just one topic but examined the breadth of medical librarianship. This expansive lecture revealed a lecturer with broad interests and insights. Also important, it revealed a presenter with a social conscience and an exemplar of the profession. He was a reluctant, feeling-not-worthy Doe lecturer in 1977 at what he considered the “nexus of the gathering of information and transfer of knowledge.” It is so apparent, to these authors in this look back forty-two years later, that Erich’s scientific inquiry of medical librarianship was absolutely on point to know changes in resources and services, technology, education, and the knowledgebase would have such a future impact on health sciences librarianship. Finally, Erich’s prediction of a talented pool of hospital librarians and women striving for equality would make such a difference in improving health.

## 

**Wayne J. Peay, FMLA,** waynejpeay@gmail.com, Librarian Emeritus, Spencer S. Eccles Health Sciences Library, University of Utah, Salt Lake City, UT

**Helen-Ann Brown Epstein, AHIP, FMLA,** hepstein@virtua.org, Informationist, Health Sciences Library, Virtua Health, Mt. Laurel, NJ
